# Supplementary material for: Expression of miR-135b in Psoriatic Skin and Its Association with Disease Improvement
Source: Cells. 2020 Jul 2;9(7):1603. doi: 10.3390/cells9071603 (PMC7408353; doi:10.3390/cells9071603)
Supplement: Supplementary file 1 [file cells-09-01603-s001.pdf]

cells

Supplementary Data

S1. Supplementaray Material and Methods.

S1.1. Tissue miRNA Isolation, Reverse Transcription and RT-PCR.

Frozen skin samples were pulverised, and total RNA was isolated using miRNeasy mini kit (Qiagen). Quantity and quality were evaluated in Nanodrop and Agilent 2100 Bioanalyzer. In all cases only those samples with RIN > 8 were included. Ten ng of RNA were used for first-strand cDNA synthesis using Universal cDNA synthesis kit II (Exiqon). Subsequent RT-PCR was performed in triplicate using PCR ExiLENT SYBR Green (Exiqon) in a CFX384 PCR detection system (Bio-Rad).

S1.2. MiRNA Target Profiling

Sequencing reads were processed with a pipeline that used FastQC (www.bioinformatics.babraham.ac.uk/projects/fastqc) to assess read quality, and Cutadapt to trim sequencing reads, eliminating Illumina adaptor remains, and to discard those that were shorter than 30 bp. Resulting reads were aligned against a human transcriptome reference (GRCh38 assembly, release 91) and gene expression was quantified with RSEM(Li and Dewey, 2011). Raw counts were processed with an analysis pipeline that used Bioconductor package Limma (Liu *et al.*, 2015) for normalisation (using TMM method) and differential expression testing, taking into account only those genes expressed at a minimal level of 1 CPM in a number of samples equal to the number of replicates of the condition with less replicates. A blocking variable was used to define pairs of samples obtained from the same patient. Changes in gene expression were considered significant if associated to a Benjamini and Hochberg adjusted p-value < 0.05. Differentially expressed genes were further filtered by selecti.

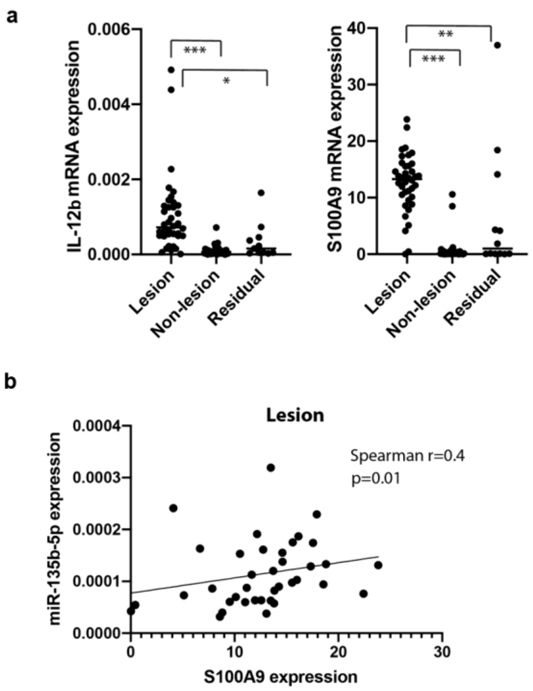

Figure S1. IL-12b and S100A9 expression in skin samples as inflammatory markers. (**a**) mRNA expression of IL-12b and S100A9 in skin samples of psoriatic patients (lesion, non-lesion and residual lesion). Data correspond to the relative levels respect to the expression of GAPDH. (**b**) Positive correlation between the levels of miR-135b-5p in and the expression of S100A9 in psoriatic skin lesions. Data were analysed using Spearman test.

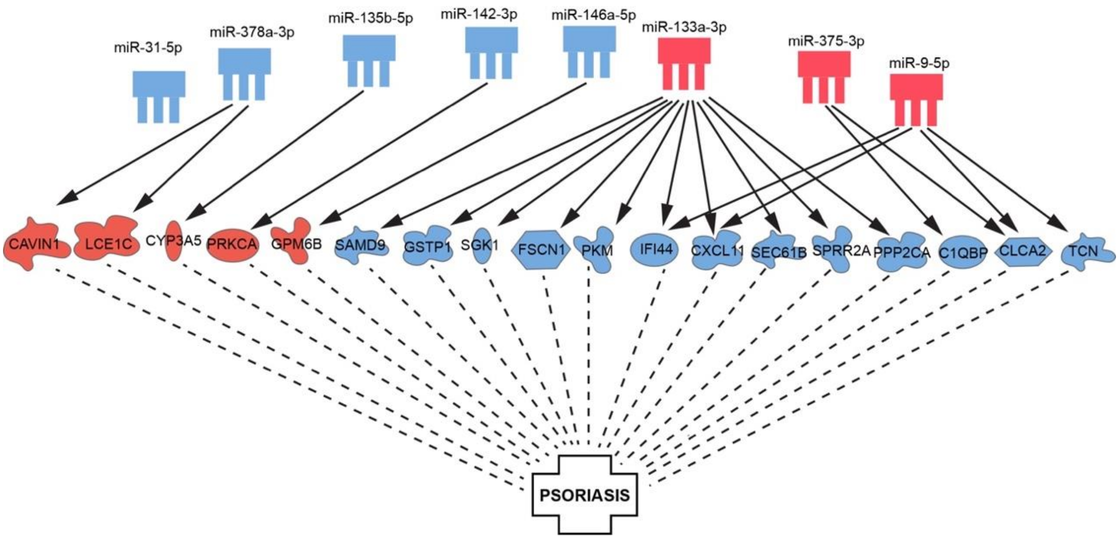

Figure S2. Interaction Network of miRNAs and target mRNAs associated with Psoriasis. Networks showing interactions between differentially expressed miRNAs and differentially expressed messenger RNA targets associated with psoriasis. Interactions were identified using IPA tool "microRNA Target Filter" of Qiagen's Ingenuity Pathway Analysis. Interacting pairs were filtered to keep only those with anti-correlated expression. Only targets experimentally observed or predicted with high confidence were included. Up-regulated molecules are shown in blue and down-regulated ones in red.

Table S1. Differentially expressed miRNAs in lesional, non-lesional psoriatic skin and healthy skin.

miRNA ID	FC[a]	*p*-val[a]	FC[b]	*p*-val[b]	FC[c]	*p*-val[c]
**hsa-mir-31**	11.460	**0.0001**	2.219	0.1779	-5.163	**0.0037**
**hsa-mir-9-5p**	"-inf"	**0.0045**	"-inf"	**0.0202**	2.437	0.3574
**hsa-mir-378a**	4.163	**0.0002**	1.984	0.0821	2.097	**0.0282**
**hsa-mir-378c**	3.766	**0.0017**	1.335	0.5354	-2.820	**0.0074**
**hsa-mir-135b**	3.956	**0.0039**	1.771	0.0876	-2.233	0.1647
**hsa-mir-33b**	3.794	**0.0046**	4.276	**0.0041**	1.1268	0.8742
**hsa-mir-375**	-5.721	**0.0060**	-2.777	0.0624	2.059	0.2674
**hsa-mir-4772**	3.799	**0.0109**	2.279	0.0561	-1.666	0.4085
**hsa-mir-454**	2.457	**0.0130**	1.499	0.3140	-1.638	0.1091
**hsa-mir-186**	-2.652	**0.0156**	-2.055	0.0758	1.290	0.445
**hsa-mir-365a**	-2.656	**0.0175**	-1.997	0.0938	1.329	0.4097
**hsa-mir-365b**	-2.581	**0.0203**	-1.889	0.1153	1.366	0.3847
**hsa-mir-30c-2**	-2.234	**0.0221**	-1.225	0.3681	1.824	0.1298
**hsa-mir-320b-1**	-5.897	**0.0226**	-2.821	0.1939	2.090	0.2395
**hsa-mir-18b**	4.916	**0.0228**	3.361	**0.0362**	-1.462	0.7102
**hsa-mir-26a-1**	-2.347	**0.0307**	-1.547	0.2061	1.516	0.3076
**hsa-mir-202**	-2.789	**0.0316**	-2.367	0.1297	1.178	0.4687
**hsa-mir-142**	2.684	**0.0326**	1.243	0.6770	-2.158	0.0672
**hsa-mir-3687**	-10.245	**0.0339**	-2.210	0.2952	4.634	0.1849
**hsa-mir-320b-2**	-3.108	**0.0344**	-2.621	**0.0273**	1.185	1
**hsa-mir-1307**	2.497	**0.0390**	2.035	0.0798	-1.227	0.6602
**hsa-mir-320a**	-3.174	**0.0438**	-2.035	0.2446	1.559	0.3141
**hsa-mir-1468**	-3.780	**0.0482**	-3.130	**0.0388**	1.207	1
**hsa-mir-615**	-1.423	0.8773	3.822	**0.0090**	5.442	**0.0052**
**hsa-mir-133a-2**	-2.084	0.1979	2.562	0.1359	5.341	**0.0064**
**hsa-mir-551b**	1.4294	0.3447	-1.597	0.1742	-2.283	**0.0223**
**hsa-mir-3687**	1.657	0.4717	6.593	**0.0030**	3.979	**0.0256**
**hsa-mir-3145**	10.3349	0.2110	inf	**0.0025**	inf	**0.0403**
**hsa-mir-4423**	1.893	0.0992	-1.258	0.5818	-2.381	**0.0273**
**hsa-mir-21**	2.434	0.2828	5.328	**0.0010**	2.188	**0.0306**
**hsa-mir-504**	-2.132	0.0745	1.470	0.8375	3.135	**0.0372**
**hsa-mir-125b**	-2.341	0.0982	1.026	0.7754	2.404	**0.0395**
**hsa-mir-374b**	-1.628	0.2777	1.375	0.3396	2.239	**0.0396**
**hsa-mir-4687**	-1.135	0.9760	3.238	0.0613	3.677	0.0553
**hsa-mir-934**	-8.436	0.0663	-31.291	**0.0020**	-3.709	0.1355
**hsa-mir-3615**	1.268	0.2911	2.817	**0.0098**	2.221	0.1215
**hsa-mir-122**	-7.776	0.0766	-11.719	**0.0163**	-1.507	0.4682
**hsa-mir-17**	1.975	0.1671	2.085	**0.0253**	1.055	0.4461
**hsa-mir-376a-2**	1.805	0.3578	3.066	**0.0257**	1.698	0.2085
**hsa-mir-1304**	2.228	0.2281	3.522	**0.0268**	1.580	0.3448
**hsa-mir-376a-1**	2.428	0.1821	2.839	**0.0297**	1.168	0.4614
**hsa-mir-296**	-2.003	0.1878	-2.400	**0.0336**	-1.198	0.4471
**hsa-mir-1228**	1.225	0.3524	3.987	**0.0337**	3.254	0.2118
**hsa-mir-224**	1.949	0.1184	2.045	**0.0349**	1.049	0.6413
**hsa-mir-1468**	-3.780	**0.0482**	-3.130	**0.0388**	1.207	1
**hsa-mir-3622a**	-3.532	0.0658	-3.275	**0.0395**	1.078	0.8768
**hsa-mir-4520a**	-2.271	0.2600	-4.489	**0.0447**	-1.654	0.3732
**hsa-mir-132**	1.344	0.4658	2.125	**0.0451**	1.580	0.2158
**hsa-mir-496**	1.5690	0.6252	7.634	**0.0453**	4.865	0.1267
**hsa-mir-1910**	10.2481	0.1410	10.382	**0.0487**	1.013	0.6984
**hsa-mir-487b**	1.255	0.6141	2.073	**0.0488**	1.651	0.1508

[a] Lesional skin vs non-lesional skin, [b] Lesional skin vs healthy, [c] Non-lesional skin vs healthy. *p* values correspond to Bonferroni *p* adjusted. FC, fold change.

Table S2. microRNAs selected from Next Generation Sequencing data.

ID	Fold Change[a,b,c]	*p*-val
**hsa-mir-31-5p**	11,4607802 [a]	0,000108979
**hsa-mir-378a**	4,16345336 [a]	0,000259515
**hsa-mir-135b-5p**	3,956695567 [a]	0,003991344
**hsa-mir-9-5p**	"-inf" [a]	0,004577766
**hsa-mir-375**	-5,721231656 [a]	0,006054568
**hsa-mir-33b**	4,27637697 [c]	0,004110427
**hsa-mir-3145**	inf [c]	0,00258317
**hsa-mir-133a-3p**	5,341909193 [b]	0,006448522
**hsa-mir-3687**	6,593690863 [c]	0,003087092
**hsa-mir-615**	5,442890822 [b]	0,005291634
**hsa-mir-934**	-31,29178488 [c]	0,002017792
**hsa-mir-142-3p**	2,68[a]	0,032614484

[a] fold change lesional/non-lesional skin, [b] fold change non-lesional/healthy skin, [c] fold change lesional/healthy skin.

Table 3. Demographic and clinical characteristics.

	**Total *n* = 44**
**Age (years),** mean±SD	49.9±15.1
**<45**	*n* = 16
**45-65**	*n* = 20
**>65**	*n* = 8
**Sex (fem/male)**	20/24
**Smoking**	14 (31.8)
**Hypertension**	12 (27.2)
**Diabetes**	7 (15.9)
**Dyslipidaemia**	7 (15.9)
**Psoriatic arthritis**	11 (25)
**Treatment (Biol/C-Syst)**	33/11

Data correspond to "*n*" (%). Abbreviations. Biol: biological treatment, C-Syst: conventional systemic treatments.

Table S4. PASI after 3 months of treatment with biological therapy in those patients with a second biopsy ($n = 15$).

PASI	anti-IL-17	anti-IL-12/IL-23	anti-TNF-a	Total
**50**	0	2	0	2
**75**	0	0	1	1
**90**	2	5	0	7
**100**	1	1	3	5

Number of patients per group.

Table S5. Univariate logistic regression to compare different levels of improvement and a set of relevant clinical variables.

	OR (95% CI)	*p* Value
**Age**	0.95 (0.91-1.99)	0.04
**Smoking**	2.2 (0.50-9.89)	0.29
**BMI**	0.98 (0.93-1.03)	0.49
**Treatment**		
* anti-IL12/IL23	0.29 (0.05-1.44)	0.131
anti-TNFa	0.53 (0.07-3.6)	0.521
**DM**	2.92 (0.51-16.1)	0.22
**NL miRNA-146a**	1.70 (0.94-3.06)	0.076
**L miRNA-135b**	3.49 (1.18-10.25)	0.023

*reference value anti-IL17 treatment. Only miRNAs with $p < 0.1$ are shown.
